# 

**Published:** 1901-12-28

**Authors:** 


### NOTICE TO CORRESPONDENTS.

All MSS., Letters, Books for Review, and other matters intended for the Editor should be addressed The Editor, "The Hospital," The Hospital Building, 28 & 29 Southampton Street, Strand, W.C.

The Editor cannot undertake to return rejected MS., even when accompanied by stamped directed envelope.

**Notice to Advertisers and Subscribers.**

All Advertisements, Orders for Copies of The Hospital, and Business Communications should be addressed to **THE MANAGER (Not to the Editor),** The Hospital, 28 & 29 Southampton Street, Strand, London, W.C.

The Cost of Subscription, post free, is as follows:—Per week, 2½d.; per quarter, 2s. 8d.; per half-year, 5s. 3d.; per annum, 10s. 6d. Foreign:—Per annum, 15s. 2d., payable, in all cases, in advance.

**NOTICE.—Vol. XXX. of "The Hospital" (April 1901, to September 1901), in handsome cloth binding can now be obtained at the office of this Journal, price, 7s. 6d. Cases for binding the Numbers contained in this Volume 1s. 6d. Index to Vol. XXX., 2½d., post free.**

**The Monthly Part for January will be ready on January 1st, 1902. Price 6d., by post 8d.**

### Scraps and Gleanings.

Queen Charlotte's Hospital has received a Grant of £100 from the Goldsmiths' Company and £10 10s. from the Salters' Company.

On the recommendation of the Public Health Committee, the Cambridge Town Council have decided to make a grant to Addenbrooke's Hospital for the year ending Michaelmas, 1902, of £125 in lieu of £5 5s. The reason for the increased grant is on account of the extra expense the hospital was put to in consequence of the late epidemic of diphtheria in the borough. This increased grant, however, represents only half of the total extra expenditure to which the hospital was put on account of this diphtheria epidemic.

At a parish meeting held at Offham, near Lewes, to consider the erection of a small-pox hospital on the borders of the parish, a resolution was passed protesting against the erection of such a hospital in the situation named on account of the position selected being on the South Downs, which are much frequented by visitors for walks, etc.

At the quarterly meeting of the Cumberland Infirmary the unsatisfactory state of the finances of the institution was strongly brought before the governors. It was pointed out that, notwithstanding the fact that £1,000 had been transferred from capital to revenue account, a sum of £865 was due to the bank; and there was also an amount of from £500 to £600 required to be expended on beds and bedding. By a resolution of the governors, a sub-committee was appointed to consider the question of a bazaar or any other mode of increasing the funds of the Infirmary.

### THE STUDENT OF MEDICINE.

### APPOINTMENTS.

**W. B. Armstrong**, M.B., C.M.Glasg., appointed Certifying Factory Surgeon for the Kirkintilloch District. **A. M. Barford**, M.D., L.R.C.P., M.R.C.S., appointed Anæsthetist to the Throat Hospital, Golden Square. **William B. Bennett**, M.R.C.S.Eng., L.R.C.P.Lond., appointed Honorary Surgeon to the St. George's Hospital for Diseases of the Skin, Liverpool. **H. A. Burridge**, M.R.C.S., L.R.C.P.Lond., appointed District Medical Officer for the Brixton District of the Lambeth Union. **George Lenthal Cheatle**, C.B., F.R.C.S., appointed Surgeon to the Italian Hospital, Queen's Square. **John Cecil Clayton**, M.R.C.S.Eng., L.R.C.P.Lond., appointed House Surgeon to the Bristol Eye Hospital. **H. Davis, jun.**, L.R.C.P.Lond., M.R.C.S.Eng., appointed Medical Officer of Health for the Callington Urban District. **Lieutenant-Colonel Andrew Deane**, M.D., F.R.C.S.I., I.M.S.(Retired), appointed Superintendent to the Royal Victoria Hospital, Belfast. **R. N. de Beauvais**, L.S.A., appointed District Medical Officer of the Sleaford Union. **Wheelton Hind**, M.D.Lond., F.R.C.S., appointed Senior House Surgeon to the North Staffordshire Infirmary and Eye Hospital, Stoke-on-Trent. **William Hunter**, M.B., C.M.Aberd., appointed Government Bacteriologist to the Colony of Hong Kong. **S. E. Johnston**, M.B., C.M.Edin., appointed Assistant Resident Medical Officer to St. Mary's Hospital for Sick Children, Plaistow. **Sydney Kent**, M.B., B.C.Camb., appointed Medical Officer and Public Vaccinator for the Bexhill District of the Battle Union. **E. M. Knott**, M.R.C.S., L.R.C.P.Lond., appointed Medical Officer of Health for the Brixworth Rural District. **William M'Lorinan**, L.R.C.P., L.R.C.S.Irel., appointed Medical Office for the Waterfoot Dispensary District of the Larne Union. **J. A. Parsons**, M.D., appointed District Medical Officer of the Stamford Union. **J. A. W. Pereira**, M.R.C.S., L.R.C.P.Lond., appointed Medical Officer to the Exeter Union Workhouse. **Charles Porter**, M.B., C.M., B.Sc.(Public Health)Edin., appointed Assistant to the Medical Officer of Health, Leith. **F. C. Rogers**, F.R.C.S.E., L.R.C.P.E., appointed Medical Officer of Health to the Erith District Council. **W. D. Spanton**, F.R.C.S., appointed Honorary Consulting to the North Staffordshire Infirmary and Eye Hospital, Stoke-on-Trent. **H. Stansfield**, M.B., Ch.B.Vict., appointed Medical Officer of Health to the Clayton District Council. **D. A. Welsh**, M.D.Edin., B.Sc., appointed Professor of Pathology in the University of Sydney. **Howard S. Willson**, B.A., M.B.Cantab., D.P.H., appointed Demonstrator of Pathology and Bacteriology in King's College, London.

